# Factors associated with pre-treatment HIV RNA: application for the use of abacavir and rilpivirine as the first-line regimen for HIV-infected patients in resource-limited settings

**DOI:** 10.1186/s12981-017-0151-1

**Published:** 2017-05-05

**Authors:** Sasisopin Kiertiburanakul, David Boettiger, Oon Tek Ng, Nguyen Van Kinh, Tuti Parwati Merati, Anchalee Avihingsanon, Wing-Wai Wong, Man Po Lee, Romanee Chaiwarith, Adeeba Kamarulzaman, Pacharee Kantipong, Fujie Zhang, Jun Yong Choi, Nagalingeswaran Kumarasamy, Rossana Ditangco, Do Duy Cuong, Shinichi Oka, Benedict Lim Heng Sim, Winai Ratanasuwan, Penh Sun Ly, Evy Yunihastuti, Sanjay Pujari, Jeremy L. Ross, Matthew Law, Somnuek Sungkanuparph, on behalf of the TREAT Asia HIV Observational Databases (TAHOD)

**Affiliations:** 1 Department of Medicine, Faculty of Medicine Ramathibodi Hospital, Mahidol University, 270 Rama VI Road, Bangkok, 10400 Thailand; 2 The Kirby Institute, University of New South Wales, Sydney, Australia; 3 Tan Tock Seng Hospital, Singapore, Singapore; 4 National Hospital of Tropical Diseases, Hanoi, Vietnam; 5 Department of Medicine, Faculty of Medicine, Udayana University & Sanglah Hospital, Bali, Indonesia; 6 HIV-NAT/Thai Red Cross AIDS Research Centre, Bangkok, Thailand; 7 Taipei Veterans General Hospital, Taipei, Taiwan; 8 Queen Elizabeth Hospital, Hong Kong, China; 9 Research Institute for Health Sciences, Chiang Mai, Thailand; 10 University Malaya Medical Centre, Kuala Lumpur, Malaysia; 11 Chiangrai Prachanukroh Hospital, Chiang Rai, Thailand; 12 Beijing Ditan Hospital, Capital Medical University, Beijing, China; 13 Division of Infectious Diseases, Department of Internal Medicine, Yonsei University College of Medicine, Seoul, South Korea; 14 Chennai Antiviral Research and Treatment Clinical Research Site (CART CRS), YRGCARE Medical Centre, VHS, Chennai, India; 15 Research Institute for Tropical Medicine, Manila, Philippines; 16 Bach Mai Hospital, Hanoi, Vietnam; 17 National Center for Global Health and Medicine, Tokyo, Japan; 18 Hospital Sungai Buloh, Sungai Buloh, Malaysia; 19 Faculty of Medicine, Siriraj Hospital, Mahidol University, Bangkok, Thailand; 20 National Center for HIV/AIDS, Dermatology & STDs, University of Health Sciences, Phnom Penh, Cambodia; 21 Faculty of Medicine Universitas Indonesia, Dr. Cipto Mangunkusumo General Hospital, Jakarta, Indonesia; 22 Institute of Infectious Diseases, Pune, India; 23 TREAT Asia, amfAR, The Foundation for AIDS Research, Bangkok, Thailand

**Keywords:** Abacavir, HIV RNA, Model, Prediction, Rilpivirine

## Abstract

**Background:**

Abacavir and rilpivirine are alternative antiretroviral drugs for treatment-naïve HIV-infected patients. However, both drugs are only recommended for the patients who have pre-treatment HIV RNA <100,000 copies/mL. In resource-limited settings, pre-treatment HIV RNA is not routinely performed and not widely available. The aims of this study are to determine factors associated with pre-treatment HIV RNA <100,000 copies/mL and to construct a model to predict this outcome.

**Methods:**

HIV-infected adults enrolled in the TREAT Asia HIV Observational Database were eligible if they had an HIV RNA measurement documented at the time of ART initiation. The dataset was randomly split into a derivation data set (75% of patients) and a validation data set (25%). Factors associated with pre-treatment HIV RNA <100,000 copies/mL were evaluated by logistic regression adjusted for study site. A prediction model and prediction scores were created.

**Results:**

A total of 2592 patients were enrolled for the analysis. Median [interquartile range (IQR)] age was 35.8 (29.9–42.5) years; CD4 count was 147 (50–248) cells/mm^3^; and pre-treatment HIV RNA was 100,000 (34,045–301,075) copies/mL. Factors associated with pre-treatment HIV RNA <100,000 copies/mL were age <30 years [OR 1.40 vs. 41–50 years; 95% confidence interval (CI) 1.10–1.80, p = 0.01], body mass index >30 kg/m^2^ (OR 2.4 vs. <18.5 kg/m^2^; 95% CI 1.1–5.1, p = 0.02), anemia (OR 1.70; 95% CI 1.40–2.10, p < 0.01), CD4 count >350 cells/mm^3^ (OR 3.9 vs. <100 cells/mm^3^; 95% CI 2.0–4.1, p < 0.01), total lymphocyte count >2000 cells/mm^3^ (OR 1.7 vs. <1000 cells/mm^3^; 95% CI 1.3–2.3, p < 0.01), and no prior AIDS-defining illness (OR 1.8; 95% CI 1.5–2.3, p < 0.01). Receiver-operator characteristic (ROC) analysis yielded area under the curve of 0.70 (95% CI 0.67–0.72) among derivation patients and 0.69 (95% CI 0.65–0.74) among validation patients. A cut off score >25 yielded the sensitivity of 46.7%, specificity of 79.1%, positive predictive value of 67.7%, and negative predictive value of 61.2% for prediction of pre-treatment HIV RNA <100,000 copies/mL among derivation patients.

**Conclusion:**

A model prediction for pre-treatment HIV RNA <100,000 copies/mL produced an area under the ROC curve of 0.70. A larger sample size for prediction model development as well as for model validation is warranted.

## Background

Antiretroviral therapy (ART) for the treatment of human immunodeficiency virus (HIV) infection has dramatically reduced HIV-associated morbidity and mortality and has transformed HIV infection into a manageable chronic condition [1, 2]. Furthermore, early ART is highly effective in preventing HIV transmission to sexual partners [3]. More than 25 antiretroviral drugs (ARV) in 6 classes are approved for treatment of HIV infection [4]. Selection of an ARV regimen should be individualized on the basis of efficacy, adverse effects, pill burden, dosing frequency, drug–drug interactions, comorbid conditions, and cost [4, 5].

The initial ARV regimen for a treatment-naïve HIV-infected patient generally consists of 2 nucleoside/nucleotide reverse transcriptase inhibitors, usually abacavir (ABC) plus lamivudine (3TC) or tenofovir disoproxil fumarate plus emtricitabine (TDF/FTC), plus a drug from 1 of 3 drug classes: an integrase strand transfer inhibitor, a non-nucleoside reverse transcriptase inhibitor (NNRTIs), or a boosted protease inhibitor [4, 5]. ABC is usually preferred over TDF for individuals with chronic kidney disease and/or those at risk of osteoporosis and fractures [4, 5]. However, ABC is recommended for patients who are HLA-B*5701 allele negative and have a pre-treatment HIV RNA <100,000 copies/mL [6], except when used with dolutegravir (DTG) and 3TC in the same regimen [4, 5].

Rilpivirine (RPV) is a recently approved NNRTI available at relatively low cost in Thailand (7 USD per month) and other countries. The advantages of RPV are once-daily dosing and very small pill size. In addition, RPV is associated with fewer treatment discontinuations for central nervous system adverse effects, fewer lipid effects, and fewer rashes when compared with efavirenz (EFV) [7, 8]. Nevertheless, RPV has a higher rate of virological failure when compared to EFV, especially in the first 48 weeks of treatment [7]. RPV is thus recommended as an alternative option for treatment naïve HIV-infected patients with a pre-treatment HIV RNA <100,000 copies/mL and CD4 count >200 cells/mm^3^ [4, 5].

Testing of HIV RNA levels is recommended during initial patient visits by treatment guidelines in developed countries [4, 5]. In resource-limited settings, pre-treatment HIV RNA is not routinely performed and not widely available [9, 10]. This limits the use of ABC and RPV as a component of the first-line ARV regimen. If a clinical prediction tool based on routinely collected data could accurately predict whether pre-treatment HIV RNA was <100,000 copies/mL, this could be applied into clinical practice. The aims of this study are to determine factors associated with pre-treatment HIV RNA <100,000 copies/mL and to construct prediction tools that predict a pre-treatment HIV RNA <100,000 copies/mL. This prediction tool might support the use of ABC and RPV as part of first-line regimens for selected treatment-naïve HIV-infected individuals in resource-limited settings with limited access to HIV RNA testing.

## Patients and methods

Our study population consisted of HIV-infected patients enrolled in the TREAT (Therapeutics Research, Education, and AIDS Training) Asia HIV Observational Database (TAHOD). The characteristics of this cohort have been described previously. Briefly, TAHOD is a prospective multi-center, observational study of patients with HIV and aims to assess HIV disease natural history in treated and untreated patients in the Asia and Pacific region [11]. We included patients enrolled in the cohort from 23 clinical sites throughout 13 countries in the Asia Pacific region since September 2003. The date of data censoring for the analysis of this study was 31 March 2015.

HIV-infected adults enrolled in TAHOD were eligible if they had an HIV RNA measurement documented at or around the time of ART initiation (pre-treatment HIV RNA). The window period of pre-treatment HIV RNA measurement was between 3 months prior to 1 day after the date of starting ART. ART was defined as a regimen containing ≥3 ARVs. Those exposed to mono or dual therapy prior to starting combination ART were excluded. Baseline was defined as the date of ART initiation. At baseline, co-variables included age, sex, HIV exposure, hepatitis B and C serology (ever positive), time since diagnosis of HIV infection, HIV subtype, and AIDS diagnosis prior to baseline. The window period of the following co-variables was between 3 months prior to 3 months after the date of ART initiation; body mass index (BMI), anemia (hemoglobin <13 g/dL for men, <12 g/dL for women), total lymphocyte count, CD4 count, CD8 count, CD4:CD8 ratio, and syphilis serology [Rapid plasma reagin (RPR), Venereal Disease Research Laboratory (VDRL) or *Treponema pallidum* particle agglutination assay (TPHA)].

### Statistical analysis

The dataset was randomly split into a derivation data set (containing data from 75% of all eligible patients) and validation data set (containing data from 25% of all eligible patients) using the PROC SURVEYSELECT command in SAS version 9.4 (SAS Institute Inc., Cary, North Carolina, USA). The study endpoint was pre-treatment HIV RNA <100,000 copies/mL. Factors associated with this endpoint were evaluated by logistic regression adjusted for study site. Co-variables were considered for inclusion in the multivariate model if one or more categories exhibited a p-value <0.1. They were retained in the multivariate model if one or more categories exhibited a p-value <0.05. Missing categories, where present, were included in all models but odds ratios (OR) were not shown.

Prediction scores were created by multiplying the OR for each multivariate co-variable category by 10 and subtracting 1 [12]. Scores were rounded to the nearest 0.5 points. Some categories among the variables including in the multivariate model gave similar OR and were therefore collapsed together for the prediction tool.

The discrimination was evaluated using the area under the receiver-operator characteristic (AUROC) curve [13]. We used data of patients that had data available on all variables including in the prediction model. The optimum cut-off point for the score was evaluated by sensitivity, specificity, positive predictive value, and negative predictive value. Stata version 14.1 (StataCorp, College Station, Texas, USA) was used for all statistical analysis.

## Results

A total of 2592 patients were included in our derivation analysis. Median [interquartile range (IQR)] age was 35.8 (29.9–42.5) years, 56.2% had heterosexual HIV exposure, median (IQR) BMI was 21.1 (19.0–23.4) kg/m^2^, median duration of HIV diagnosis was 4.3 (1.4–29.2) months, and 34.5% had prior AIDS-defining illness. Median CD4 count was 147 (50–248) cells/mm^3^ and median pre-treatment HIV RNA was 100,000 (34,045–301,075) copies/mL. For other laboratory investigations, 49.3% had anemia, 10.8% had positive HBsAg, 8.3% had positive anti-HCV, 19.6% had positive syphilis serology, and 75.1% had HIV infection with CRF01_AE subtype. Baseline characteristics of the patients are shown in Table 1.

**Table 1:** Baseline characteristics of 2592 HIV-infected patients

Baseline characteristics	Value^a^
Median (IQR) age, years	35.8 (29.9–42.5)
Male	1883 (72.6)
HIV exposure	
Heterosexual	1456 (56.2)
Homosexual	778 (30.0)
Intravenous drug use	93 (3.6)
Other	265 (10.2)
Median (IQR) body mass index, kg/m^2^	21.1 (19.0–23.4)
Missing	683 (26.4)
Anemia	
No, n (% tested)	1208 (50.7)
Yes, n (% tested)	1176 (49.3)
Unknown	208 (8.0)
Hepatitis B surface antigen	
Negative, n (% tested)	1925 (89.2)
Positive, n (% tested)	232 (10.8)
Unknown	435 (16.8)
Hepatitis C antibody	
Negative, n (% tested)	1844 (91.7)
Positive, n (% tested)	168 (8.3)
Unknown	580 (22.4)
Syphilis serology	
Negative, n (% tested)	825 (80.4)
Positive, n (% tested)	201 (19.6)
Unknown	1566 (60.4)
Median (IQR) duration of HIV diagnosis, months	4.3 (1.4–29.2)
Missing	29 (1.1)
HIV subtype	
CRF01_AE, n (% tested)	796 (75.1)
B, n (% tested)	173 (16.3)
Other, n (% tested)	91 (8.6)
Unknown	1532 (59.1)
Median (IQR) HIV RNA, copies/mL	100,000 (34,045–301,075)
Median (IQR) CD4 count, cells/mm^3^	147 (50–248)
Missing	106 (4.1)
Median (IQR) CD8 count, cells/mm^3^	753 (485–1103)
Missing	1268 (48.9)
Median (IQR) CD4:CD8 ratio	0.19 (0.09–0.32)
Missing	1268 (48.9)
Median (IQR) total lymphocyte count, cells/mm^3^	1472 (1000–2005)
Missing	286 (11.0)
Prior AIDS illness	
No	1698 (65.5)
Yes	894 (34.5)

*IQR* interquartile range

^a^ Values are n (% total) unless otherwise specified

Factors that statistically significantly associated with pre-treatment HIV RNA <100,000 copies/mL in the derivation patients by multivariate logistic regression, were age <30 years [OR 1.40 vs. 41–50 years; 95% confidence interval (CI) 1.10–1.80, p = 0.01], body mass index >30 kg/m^2^ (OR 2.4 vs. <18.5 kg/m^2^; 95% CI 1.1–5.1, p = 0.02), anemia (OR 1.70; 95% CI 1.40–2.10, p < 0.01], CD4 count >350 cells/mm^3^ (OR 3.9 vs. <100 cells/mm^3^; 95% CI 2.0–4.1, p < 0.01), total lymphocyte count >2000 cells/mm^3^ (OR 1.7 vs. <1000 cells/mm^3^; 95% CI 1.3–2.3, p < 0.01), and no prior AIDS-defining illness (OR 1.8; 95% CI 1.5–2.3, p < 0.01) (Table 2).

**Table 2:** Factors associated pre-treatment HIV RNA <100,000 copies/mL in derivation population

Factors	Number of patients	Patients (% total) with HIV RNA <100,000 copies/mL	Univariate OR (95% CI)	p-value	Multivariate OR (95% CI)	p- value
Years of age^b^						
≤30	656	360 (54.9)	1.6 (1.2–2.0)	<0.01	1.4 (1.1–1.8)	0.01
31–40	1057	514 (48.6)	1.2 (0.9–1.4)	0.15	1.1 (0.9–1.4)	0.40
41–50	600	273 (45.5)	1.0		1.0	
>50	279	131 (47.0)	1.0 (0.8–1.4)	0.85	1.0 (0.7–1.4)	0.96
Sex^b^						
Male	1883	908 (48.2)	1.0			
Female	709	370 (52.2)	1.2 (1.0–1.5)	0.02		
HIV exposure						
Heterosexual	1456	688 (47.3)	1.0			
Homosexual	778	422 (54.2)	1.3 (1.0–1.6)	0.03		
Intravenous drug use	93	41 (44.1)	0.9 (0.6–1.4)	0.54		
Other	265	127 (47.9)	0.9 (0.7–1.3)	0.70		
Body mass index (kg/m^2^)^b^						
<18.5	366	134 (36.6)	1.0		1.0	
18.5–24.9	1289	648 (50.3)	1.7 (1.3–2.2)	<0.01	1.3 (1.0–1.7)	0.07
25.0–29.9	213	112 (52.6)	1.8 (1.3–2.6)	<0.01	1.2 (0.8–1.7)	0.47
≥30.0	41	29 (70.7)	4.1 (2.0–8.4)	<0.01	2.5 (1.2–5.2)	0.02
Unknown	683	355 (52.0)	–		–	
Anemia^b^						
No	1208	741 (61.3)	2.7 (2.3–3.3)	<0.01	1.7 (1.4–2.1)	<0.01
Yes	1176	442 (37.6)	1.0		1.0	
Unknown	208	95 (45.7)	–		–	
Hepatitis C antibody						
Negative	1844	916 (49.7)	1.0			
Positive	168	73 (43.5)	0.8 (0.6–1.1)	0.15		
Unknown	580	289 (49.8)	–			
Month since HIV diagnosis						
<6	1384	614 (44.4)	1.0			
6–18	338	189 (55.9)	1.6 (1.3–2.1)	<0.01		
>18	841	461 (54.8)	1.5 (1.3–1.8)	<0.01		
Unknown	29	14 (48.3)	–			
CD4 count (cells/mm^3^)^b^						
≥350	219	147 (67.1)	4.8 (3.4–6.7)	<0.01	2.9 (2.0–4.1)	<0.01
200–349	694	456 (65.7)	4.2 (3.4–5.3)	<0.01	2.7 (2.1–3.4)	<0.01
100–199	617	308 (49.9)	2.1 (1.7–2.6)	<0.01	1.6 (1.2–2.0)	<0.01
<100	956	318 (33.3)	1.0		1.0	
Unknown	106	49 (46.2)	–		–	
Total lymphocyte count (cells/mm^3^)^a^						
≥2000	593	325 (54.8)	2.9 (2.2–3.7)	<0.01	1.7 (1.3–2.3)	
1500–1999	529	303 (57.3)	2.9 (2.3–3.7)	<0.01	1.8 (1.4–2.4)	
1000–1499	627	331 (52.8)	2.3 (1.8–2.9)	<0.01	1.6 (1.3–2.1)	<0.01
<1000	557	180 (32.3)	1.0		1.0	
Unknown	286	139 (48.6)	–		–	
Prior AIDS-defining illness^a^						
None known	1698	987 (58.1)	3.0 (2.5–3.6)	<0.01	1.8 (1.5–2.3)	<0.01
Yes	894	291 (32.6)	1.0		1.0	

*OR* odds ratio, *CI* confidence interval

^a^ Multivariate result shows effect size when replacing CD4 count

^b^ Included in the final model

Clinical prediction tool scores for pre-treatment HIV RNA <100,000 copies/mL are shown in Table 3. Scores were +3.5 for age <30 years, +2.5 for BMI of 18.5–29.9 kg/m^2^ or +14.5 for BMI of >30 kg/m^2^, +7.0 for non-anemia, +17.0 for CD4 count >200 cells/mm^3^ or +5.5 for 100–199 cells/mm^3^, and +8.5 for no prior AIDS-defining illness. The possible maximum score was 50.5.

**Table 3:** Clinical prediction tool scores for each variable for pre-treatment HIV RNA <100,000 copies/mL

Variables	Score
Age ≤30 years	+3.5
Age >30 years	0
Body mass index <18.5 kg/m^2^	0
Body mass index 18.5–29.9 kg/m^2^	+2.5
Body mass index ≥30 kg/m^2^	+14.5
Anemic	0
Non-anemic	+7.0
CD4 count ≥200 cells/mm^3^	+17.0
CD4 count 100–199 cells/mm^3^	+5.5
CD4 count <100 cells/mm^3^	0
No prior AIDS-defining illness	+8.5
Prior AIDS-defining illness	0
Maximum score	50.5

**Fig. 1:**
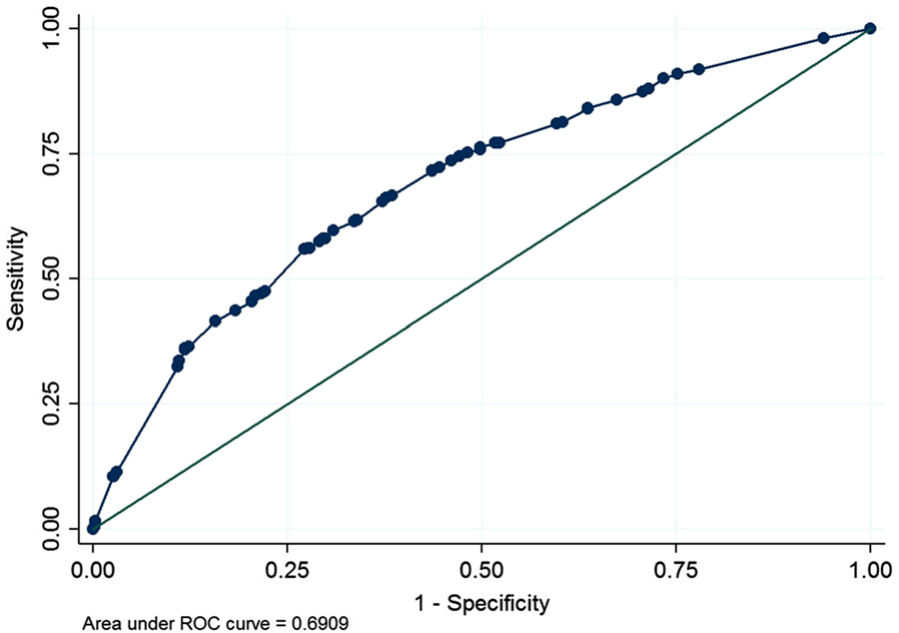
Receiver-operator characteristic curve for predicting pre-treatment HIV RNA <100,000 copies/mL among derivation patients with data on all included variables (n = 1757)

AUROC analysis was 0.70 (95% CI 0.67–0.72) among the derivation patients (Fig. 1) and 0.69 (95% CI 0.65–0.74) among validation patients.

A cut off total score >25 yielded sensitivity of 46.7 and 47.4%, specificity of 79.1 and 77.1%, positive predictive value of 67.7 and 64.2%, and negative predictive value of 61.2 and 63.0% for pre-treatment HIV RNA <100,000 copies/mL among the derivation patients and validation patients, respectively (Tables 4, 5). In contrast a cut off score >5 yielded the highest sensitivity of 91.1 and 91.9% and lowest specificity of 24.8 and 24.1% among derivation patients and validation patients, respectively (Tables 4, 5). We also conducted a sensitivity analysis using other prediction models, e.g. using total lymphocyte count instead of CD4 count and restriction analysis only among patients with CD4 count >200 cells/mm^3^, however these models did not perform better.

**Table 4:** Sensitivities, specificities, positive predictive values, and negative predictive values of clinical prediction tool for pre-treatment HIV RNA <100,000 copies/mL among derivation patients with data on all included variables (n = 1757)

CPT score	N (%)	N (%) tests avoided	Sensitivity (%)	Specificity (%)	PPV (%)	NPV (%)
>25.0	586 (37.7)	1171 (75.3)	46.7	79.1	67.7	61.2
>20.0	764 (49.1)	993 (63.8)	58.0	70.2	64.7	64.0
>15.0	1018 (65.4)	739 (47.5)	72.3	55.5	60.4	68.1
>10.0	1239 (79.6)	518 (33.3)	81.3	39.6	55.9	69.3
>5.0	1456 (93.6)	301 (19.3)	91.1	24.8	53.2	74.8

*CPT* clinical prediction tool, *PPV* positive predictive value, *NPV* negative predictive value

**Table 5:** Sensitivities, specificities, positive predictive values, and negative predictive values of clinical prediction tool for pre-treatment HIV RNA <100,000 copies/mL among validation patients with data on all included variables (n = 587)

CPT score	N (%)	N (%) tests avoided	Sensitivity (%)	Specificity (%)	PPV (%)	NPV (%)
>25.0	201 (39.5)	386 (75.8)	47.4	77.1	64.2	63.0
>20.0	264 (51.9)	323 (63.5)	61.0	68.9	62.9	67.2
>15.0	355 (69.7)	232 (45.6)	75.4	52.4	57.7	71.1
>10.0	421 (82.7)	166 (32.6)	84.6	39.4	54.6	74.7
>5.0	489 (96.1)	98 (19.3)	91.9	24.1	51.1	77.6

*CPT* clinical prediction tool, *PPV* positive predictive value, *NPV* negative predictive value

## Discussion

Plasma HIV RNA is one laboratory test used to stage HIV disease and to assist in the selection of ARV drug regimens [4, 5]. If treatment-naïve HIV-infected patients have a pre-treatment HIV RNA >100,000 copies/mL, the following regimens are not recommended; ABC/3TC with EFV or atazanavir/ritonavir (ATV/r) or raltegravir (RAL), RPV-based regimens, and darunavir/r (DRV/r) plus RAL [4, 5]. The main reason is being the higher rates of virologic failure observed in patients who received these particular drugs [7]. In addition, patients with pre-treatment HIV RNA >100,000 copies/mL or CD4 count <200 cells/μL are a subset of patients who may experience suboptimal virologic suppression if the regimen consists of ABC or PRV [5].

To our knowledge, this is the first study on prediction tool of pre-treatment HIV RNA <100,000 copies/mL in treatment-naïve HIV-infected patients that aims to facilitate the use of ABC and RPV as one of ARV in the first-line ART in resource-limited settings. We found some clinical and laboratory factors statistically significantly associated with pre-treatment HIV RNA <100,000 copies/mL. Our prediction tool of pre-treatment HIV RNA <100,000 copies/mL performed AUROC curve of 0.70. A cut off score >25 yielded the highest specificity of 79.0% for predicting pre-treatment HIV RNA <100,000 copies/mL.

Few studies focus on the association between HIV RNA levels and HIV-related outcomes. The results from some previous studies showed that HIV RNA level is rarely directly associated with the type of opportunistic infection [14] or HIV disease progression [15]. One study demonstrated a significant correlation between HIV RNA level and wasting syndrome in naïve HIV-infected patients, with HIV RNA levels in patients with wasting syndrome, significantly higher than those without the condition [16].

We also found six independent factors associated with pre-treatment HIV RNA <100,000 copies/mL: age, BMI, anemia, CD4 count, total lymphocyte count, and prior AIDS-defining illness. For example, patients with age <30 years had higher odds of 1.4 of having pre-treatment HIV RNA <100,000 copies/mL compared to patients 41–50 years old. Furthermore, patients with baseline CD4 count 100–199 cells/mm^3^ had higher odds of 1.6 of having pre-treatment HIV RNA <100,000 copies/mL compared to patients with baseline CD4 count <100 cells/mm^3^. These factors might be easily applied in the assessment of patients in resource-limited settings because they are patients’ clinical characteristics and routine baseline laboratory investigations.

The AUROC curve is a single index for measuring the performance a test and can be used to estimate the discriminating power of a test. The AUROC of a ‘perfect’ test would be 1.00, that of a useless test, 0.50 [13, 17]. The AUROC for the pre-treatment HIV RNA model applied to the derivation population was 0.70. The AUROC curve when the model was applied to the validation population was 0.69, indicating some loss of discriminating power when applied to the new population. The score >5 showed the highest sensitivity but lowest specificity. With prediction of pre-treatment HIV RNA <100,000 copies/mL, higher specificity is required to minimize false positive results. Using a score >25 for prediction of pre-treatment HIV RNA yielded specificity approximately 80% and positive predictive value almost 70% and might be more appropriate. Additional data variables and/or an increased number of the patients might be needed to improve this prediction model and enhance its performance.

This study had some limitations. First, some patients must be excluded from the regression analysis and from the prediction tool due to missing data. Second, the performance of the model described by the AUROC of 0.70 might be associated with the small sample size of the study population among derivation and validation group.

In conclusion, in situations where HIV RNA cannot be obtained prior to ART initiation due to high costs or limited availability, certain risk factors and models for predicting pre-treatment HIV RNA <100,000 copies/mL might be useful to predict pre-treatment HIV RNA and afford opportunities for ABC and RPV initiation among naïve HIV-infected patients. A larger sample size with greater data variety would be warranted for prediction model construction as well as for model validation. Pre-treatment HIV RNA should be performed before ABC and RPV initiation if it is available and affordable.

## Abbreviations

ART: antiretroviral therapy
HIV: human immunodeficiency virus
ARV: antiretroviral drugs
ABC: abacavir
3TC: lamivudine
TDF: tenofovir disoproxil fumarate
FTC: emtricitabine
NNRTI: non-nucleoside reverse transcriptase inhibitors
DTG: dolutegravir
RPV: rilpivirine
EFV: efavirenz
TREAT: Therapeutics Research, Education, and AIDS Training
TAHOD: TREAT Asia HIV Observational Database
BMI: body mass index
RPR: rapid plasma regain
VDRL: Venereal Disease Research Laboratory
TPHA: *Treponema pallidum* particle agglutination assay
OR: odds ratio
AUROC: area under the receiver-operator characteristic
IQR: interquartile range
CI: confidence interval
ATV/r: atazanavir/ritonavir
RAL: raltegravir
DRV/r: darunavir/r
CPT: clinical prediction tool
PPV: positive predictive value
NPV: negative predictive value
